# Participatory ecosystem service mapping to enhance community-based mangrove rehabilitation and management in Demak, Indonesia

**DOI:** 10.1007/s10113-018-1378-7

**Published:** 2018-07-18

**Authors:** Ekaningrum Damastuti, Rudolf de Groot

**Affiliations:** Environmental Systems Analysis Group, Wageningen University and Research, PO Box 47, 6700AA Wageningen, Netherlands

**Keywords:** Ecosystem services, Environmental change, Participatory resource mapping, GIS, Community-based mangrove management, Demak

## Abstract

Assessment of mangrove ecosystem services (ES) is essential to understand and manage the contribution of these ecosystems to the well-being of local communities. They are the primary beneficiaries but their experience, knowledge, and information are frequently ignored in ES assessment and mapping. In this study, a participatory resource mapping (PRM) approach was applied using local knowledge and experience to analyze geo-referenced information on mangrove ecosystem services. Local communities were involved from the beginning in method selection, application, evaluation, and verification. This “inclusive participatory ES mapping” was conducted in two villages (Bedono and Timbulsloko, Central Java, Indonesia) from 2014 to 2015. Participants representing different community elements were involved in the mapping process. They first created a historical map of the situation in their villages roughly between 1980 and 1999 (before rehabilitation) and then described the subsequent environmental changes. The mapping exercise also documented different mangrove resources that are utilized by communities and identified key areas, such as harvesting zones, biodiversity hotspots, erosion zones, different fishing grounds, and newly rehabilitated areas. The maps reveal that integrating PRM and indigenous geo-referenced information can elicit past and contemporary information on (changes in) ecosystem service availability and use. The results show that by involving local communities from the beginning, the participatory ES mapping can facilitate social learning, provide the foundation for the creation of social capital, and equip the community with sufficient spatial information to improve local mangrove management. The participatory ES mapping approach presented in this paper can be used as a model to support local and regional decision-making processes and to enhance community-based mangrove management in other coastal regions in Indonesia and beyond.

## Introduction

The importance of mangroves to support local livelihoods by providing ecosystem services (ESs; e.g., provision of foods, raw materials, and medicinal resources,) has been widely recognized (Chong 2007; Kusmana 2011). Recent studies also highlighted their crucial role in protecting the coastline from storms and even tsunamis (Alongi 2008; Hashim and Catherine 2013; Ilman et al. 2016). Despite their large ecological and livelihood importance, mangroves have long been the subject of human disturbance (e.g., coastal development, conversion to aquaculture, timber overharvesting, and pollution) (Alongi 2008; Sudtongkong and Webb 2008). More than 3 million hectares of mangroves worldwide disappeared within only 25 years (1980–2005) with a degradation rate of 1% per year (Mayaux et al. 2005). This degradation was mostly caused by mangrove conversion to aquaculture/agriculture, of which the majority occurred in South East Asia (Thomas et al. 2017), including Indonesia. In the 1980s, the shrimp farming boom triggered large-scale mangrove conversion to aquacultures in this country. This conversion mainly took place in Java (Ilman et al. 2016; Setyawan et al. 2003, 2004). Currently, the total mangrove area left in Java is only 45,000 ha or less than one third of its original size in 1800 (Ilman et al. 2016).

In the past three decades, increasing understanding of the environmental and livelihood importance of mangroves has stimulated various rehabilitation initiatives in Java. The earliest rehabilitation effort started in the 1960s by the State Forest Cooperation (Perhutani) (Kusmana 2014). However, the top-down strategy applied by this institution was unable to halt human encroachment into the rehabilitated areas. Exclusion of local socioeconomic issues and lack of community participation was argued to be the cause of the continued disturbance (Kusmana 2011, 2014). Therefore, Perhutani started to include local communities in its rehabilitation and management strategy. This new strategy proved to be successful in reducing human disturbance, while at the same time increasing local livelihood (Kusmana 2011). Nowadays, community participation has become the mainstream approach in mangrove rehabilitation and management applied in Java (Amri 2005; Armitage 2002; Brown et al. 2014; Datta et al. 2012; Elliott et al. 2001; Purnomo et al. 2015; Rusdianti and Sunito 2012; Setyawan et al. 2004; Sidik 2008). The Ministry of Environment and Forestry, for example, has implemented community-based mangrove rehabilitation activities in this region since 2003, covering a total area of nearly 56,000 ha (MoEF 2015; MoF 2008, 2014).

In spite of these efforts to rehabilitate the mangroves in Java, only a few studies have mapped the rehabilitated mangrove areas in this region, including Ardli and Wolff (2008), Kamal et al. (2015), Hartini et al. (2010), Saputro et al. (2009), Maryantika and Lin (2017), and Fitzastri et al. (2016). Overall, none of the existing mapping studies on mangroves in Java addressed mangrove ESs used by local communities or involved the communities in the mapping process. To improve mangrove ecosystem management, spatial information on the current state and local uses of mangrove ESs is arguably necessary for monitoring, communication, and decision making (Brown and Fagerholm 2015; Maes et al. 2012; Magris and Barreto 2010; Paudyal et al. 2015). Although national and regional policy decisions may influence local management, conservation and sustainable use of mangroves largely depends on the local communities’ attitude and their resource utilization pattern (Badola et al. 2012; Roy 2016). Moreover, local communities often better understand their surrounding environment than external experts. Therefore, integrating local knowledge and perspectives in mapping ESs is critical for their future management (Paudyal et al. 2015). Furthermore, effective mapping to support local management is only possible if the outputs can be easily understood by all users and cater for communities’ needs (Ramirez-Gomez et al. 2015).

A wide range of participatory mapping approaches for ecosystem services assessments has been applied in different countries (Brown and Fagerholm 2015; Fagerholm et al. 2012; Klain and Chan 2012; Paudyal et al. 2015; Plieninger et al. 2013; Ramirez-Gomez et al. 2015; Sherrouse et al. 2011). The practitioners used different tools in their mapping activities such as ephemeral mapping, sketch mapping, scale mapping, three-dimensional mapping, photo-voice mapping, video mapping, and digital (internet-based) mapping (Berbés-Blázquez 2012; Brown and Fagerholm 2015; Corbett 2009; McCall and Dunn 2012; Rambaldi et al. 2006b). These tools were applied through various techniques such as focus group discussions, workshops, semi-structured interviews, paper-based surveys, internet-based surveys, transect walks or ground-truthing, and the combination of two or more methods (Brown and Fagerholm 2015; Corbett 2009; McLain et al. 2013; Pocewicz et al. 2012). Each of these tools and data collection techniques has its strengths and limitations. For example, ephemeral and sketch mapping may produce rapid information at low cost, but the result is difficult to be reproduced into a geo-referenced map usable for all users, including the government bodies. On the other hand, digital internet-based mapping may provide precise and sufficient spatial data quality, but this technique requires advanced technology that is not applicable in many rural areas of developing countries like Indonesia (Corbett 2009; Paudyal et al. 2015).

To our knowledge, the use of participatory mapping to collect spatial information on mangrove ESs in Indonesia, and in Java in particular, has not been done before. Participatory mapping is essential to deal with data scarcity, to make the ES research more relevant to users (Ramirez-Gomez et al. 2015), and to improve natural resource management (Dunn 2007). Furthermore, local communities may have different interests in, and preferences for mapping activities, methods, and objects than researchers (Ramirez-Gomez et al. 2015), yet, they are rarely taken into account in decisions regarding the choice and design of participatory ES mapping studies. In our study, we therefore extended the application of workshop-based participatory resource mapping (PRM) by involving local communities in the process from the beginning to identify, map, and analyze the changes in mangrove ESs. PRM refers to a tool commonly used to acquire systematic and spatial information of resources and their utilization based on communities’ knowledge and perspectives (Mbile et al. 2003; McLain et al. 2013). We integrated the GIS tool in the process to capture, manage, store, analyze and transform the information collected through PRM into geo-referenced mangrove ES maps. We also included a dissemination workshop to communicate and increase the applicability of the resulting mangrove ES maps.

In this paper, we specifically aim to answer the following questions: (1) How has the overall mangrove landscape and locally important mangrove ecosystem services changed since the 1980s? (2) What are the factors influencing the changes of the ecosystem services? (3) How can the mapping processes and results contribute to enhance local mangrove management?

## Study area

**Fig. 1:**
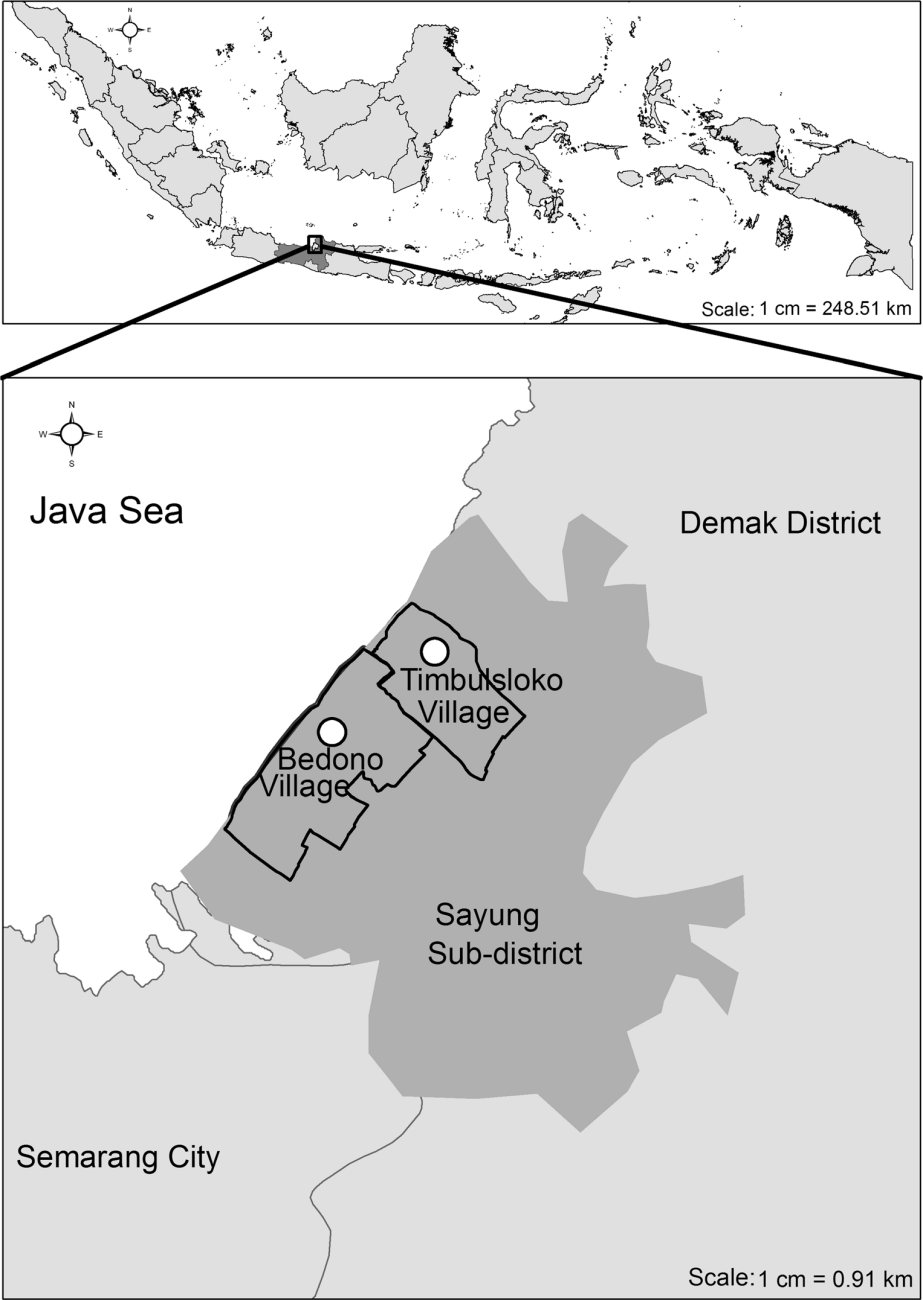
Map of study area

The Participatory ES mapping was carried out in two coastal villages, Bedono, and Timbulsloko, in Sayung sub-district, Central Java, Indonesia (see Fig. 1). These villages were selected based on the dynamic changes of this coastal area, the presence of community-based mangrove rehabilitation and management activities, and high resource use activities in and around the mangroves.

Demak district is located adjacent to the Java sea, approximately 26 km from Semarang, the capital city of Central Java. The district’s coastal areas stretch along 13 villages including Bedono and Timbulsloko in Sayung Sub-District (see Fig. 1) (Supriharto 2014). The coastal area of Demak is characterized by lowland topography elevated between 0 and 3 m above sea level (Marfai 2012; Sutikno 2015). More than 30% of these villages’ areas are used for agriculture, whereas the rest is used for settlements, yards, aquaculture, and infrastructure. In the past, the mangroves grew naturally along the villages’ seaside. However, these ecosystems were massively converted into ponds in the 1980s and nearly disappeared due to increasing coastal erosion caused by beach and harbor development of the nearby city of Semarang (Fikriyani and Mussadun 2014; Marfai 2012).

Numerous mangrove rehabilitation projects have been implemented in these villages since the late 1990s. These projects involved different actors, including local communities, government, NGOs, and private organizations. Some of the projects were successful in restoring the mangroves while engaging the communities in the management, whereas the rest failed due to high natural disturbance and lack of monitoring and maintenance. After more than 10 years of continuous planting activities, mangroves can now be seen occupying the seaside ponds and settlements with a total area of more than 160 ha. The return of mangroves in these villages provides a valuable source of livelihood that can be observed from the increase in economic activities within and around the rehabilitated areas.

## Material and methods

We used PRM approach in combination with GIS to build spatial information of both the historical and present state of the mangrove ecosystems and their utilization. We combined different tools (i.e., sketch and scale mapping) and techniques (i.e., focus group discussion, workshop, and transect walk) to gain consensual qualitative information of ESs based on local collective memories and perception. We applied an inclusive participatory approach in which the communities were involved from the beginning in method selection, application, evaluation, and verification. The overall mapping process was carried out from November 2014 to January 2015, involving 25 participants in each village.

### Preparation

The preparation covered two main activities including building good relations with the villagers and local authorities through both personal and institutional communication and preparing technical details for the mapping exercise. We lived with local families during the whole research period and used local language in our daily conversation. This enabled us to observe and participate in communities’ life and communicate our project formally and informally. We developed cooperation with the village officers and some villagers to prepare the technical details for the mapping activities, including setting up the meetings, determine the participants, and prepare the venue and equipment. We applied purposive sampling to select the participants. The number of participants involved in the mapping process was determined based on two criteria: the number of sub-villages and the number of community associations involved in mangrove rehabilitation and management. We then applied additional criteria, i.e., gender, age, and occupation to achieve a balanced representation of different elements in the community. We also added information retrieved from our observations and informal communication with villagers in the selection process to avoid the exclusion of relevant actors due to personal political interest. Regarding the mapping material, we prepared the satellite imagery of the villages retrieved from Google Earth in 2014 to be used by the participants during the mapping process.

### Mapping process

In this study, the participants were involved in the discussion and selection of the mapping process and methods to ensure they were suitable for them. We used the six common stages and methods applied in participatory mapping summarized in Corbett (2009) to guide the participants. The discussion process resulted in slightly different methods and steps applied in the two villages. The participants in Bedono prefered to start with scale mapping followed with training, ground truthing, verification, and dissemination, while in Timbulsloko, the participants were less confident and chose to sketch their village before using scale mapping.

#### Introduction

In this step, we discussed the village condition, the mangrove ecosystems, the rehabilitation activities, and the importance of maps to manage the village and mangrove rehabilitated areas. We also provided an overview of the mapping process and different mapping tools and techniques including ephemeral, sketch, and scale mapping. Ephemeral mapping refers to drawing a map on the ground using raw materials like soil, pebbles stick, and leaves. Sketch mapping means drawing a map on large sheets of paper. Scale mapping involves marking and drawing the features on a geo-coded and scaled map. The participants were then given the freedom to determine the method that will be used to map their village. The participants in Timbulsloko chose to do sketch mapping first and continued with scale mapping. The participants in Bedono preferred to do scale mapping directly.

#### Sketch and scale mapping

This step began with a discussion on the attributes that will be mapped and determine the legends for these attributes. The participants in Bedono preferred to use the example of legends provided during the meeting, whereas the participants in Timbulsloko preferred to create their legends. The participants were then split into small groups based on their sub-villages. Each small group mapped the past and present condition of their sub-villages using a scale map in Bedono and both a sketch map and scale map in Timbulsloko. The participants marked the attributes on the satellite image using colored polygons. Additional information was added using points and sticky notes. The maps from each sub-village were then combined, evaluated, and corrected by all participants.

#### Training

The participants were trained to use the Global Positioning System (GPS) tool and record the coordinate from the GPS for backup data. They also discussed the technical preparation needed for the next step (ground truthing), including the strategic time to start the activity, the vehicle needed and the person responsible for conducting the activity. Some areas in the villages, especially the mangroves, can only be reached by boat. Therefore the participants had to do the activities during the high tide.

#### Ground truthing

The ground truthing step was arranged by sub-village. Each sub-village created a small group consisting of three participants selected based on consensus among all participants. Each participant involved in this activity was given different tasks, i.e., marking, recording the coordinates, and preparing the boat and logistics needed for mapping. Each group of participants was accompanied by two facilitators who ensured that all attributes discussed in the previous meeting are marked and recorded by participants.

#### GIS processing and map verification

The data collected from the previous steps were combined and processed into geo-referenced maps. All recorded coordinates were digitized using ArcGIS 10.2. The maps were created in 1:6000 scale and printed on A0 (841 × 1189 mm) paper for verification by all participants and other stakeholders, i.e., district and sub-district government and NGOs. The maps were then revised based on their evaluation. The fifth and sixth steps were conducted iteratively to ensure that all information and input as discussed in the previous steps are included in the map.

#### Result socialization and dissemination

After two (in Bedono) to three (in Timbulsloko) verification and revision rounds, the final version of the maps was then given back to the communities through a multi-stakeholder meeting. Aside from socializing the map, this final meeting was also used to disseminate the maps to all stakeholders and discuss the possibility to improve mangrove management in these villages based on the final maps. We closed the session with a discussion and interview on the benefits of the mapping process and results for mangrove management in the two villages. After the project ended, we sent the digital version of the maps to different stakeholders involved in mangrove management in these villages (i.e., government, NGOs, and education institutions).

## Result

The mapping process facilitates the exchange of collective memories of villagers on the village condition between the 1980s and 1990s. This process also facilitates the consensus on the present (2014) condition of the village, mangrove resources, and their utilization. The spatial and narrative construction of these collective memories and consensus showed significant landscape changes in the two villages, and the factors influenced these changes.

### Spatial and narrative construction of the villages’ previous and present condition

#### Bedono village

**Fig. 2:**
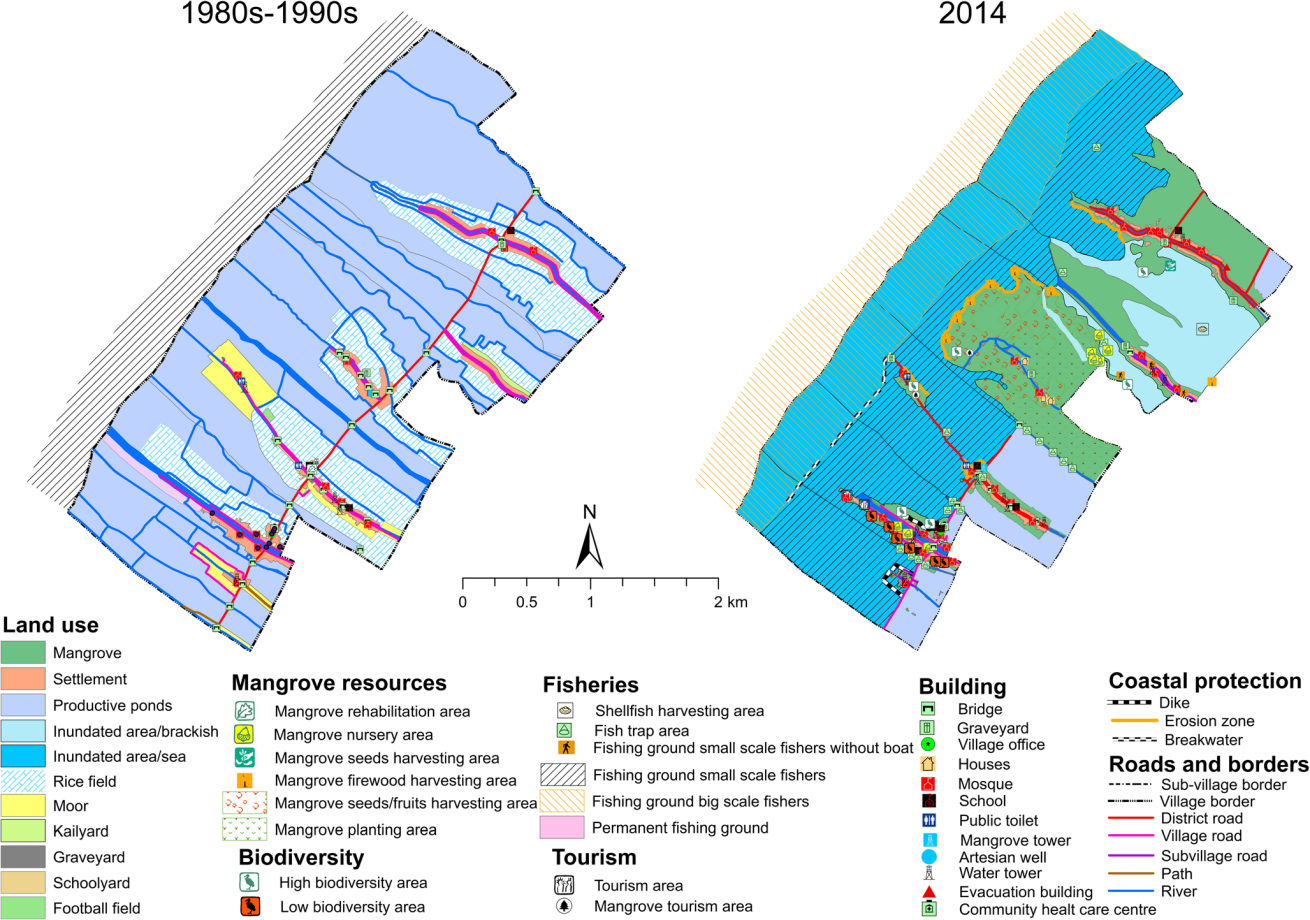
(left) Historical map (1980s to 1990s) and (right) present map (2014) of Bedono

During the mapping process, the participants confirmed that their village had lost the mangroves that used to occupy the seashore and river banks before 1980s. The main drivers for the loss were conversion to aquaculture and excessive use of mangroves for firewood. As shown in Fig. 2 (left), the village’s landscape in the 1980s was dominated by a vast area of extensive fish ponds stretched along the coast. The settlement was surrounded by productive rice fields, moors, and kailyards and located at least 2 km away from the coastline. According to the participants, the farmers started to convert their fields into fish ponds in the 1990s due to increased salinization. Furthermore, in the mid-1990s, intensive coastal erosion damaged some ponds, especially those located adjacent to the sea. Worsening coastal erosion coupled with flooding and inundation submerged hectares of ponds and forced the communities to change their occupation (Joseph et al. 2013). More than 200 households from two sub-villages, i.e., Tambaksari and Rejosari, were evacuated in 2013 (Marfai 2012). Only ten households stayed in Tambaksari and one household in Rejosari.

Various efforts to protect the village from erosion, tidal floods, and inundation had been implemented by different government institutions. These efforts included construction of breakwaters with bamboo, concrete, and stone, construction of concrete sea belt, and mangrove rehabilitation (see Fig. 2, right). The rehabilitation in Bedono started in 1999 by the Demak Environmental Office. In 2004, an international NGO named the Organization for Industrial, Spiritual and Cultural Advancement (OISCA) began rehabilitation projects in this village. At the same time, the Agricultural Office also started to implement a project entitled “National Movement for Forest and Land Rehabilitation” (GNRHL) in Bedono. Since then, various rehabilitation projects sponsored by different actors have been executed in the village.

#### Timbulsloko village

**Fig. 3:**
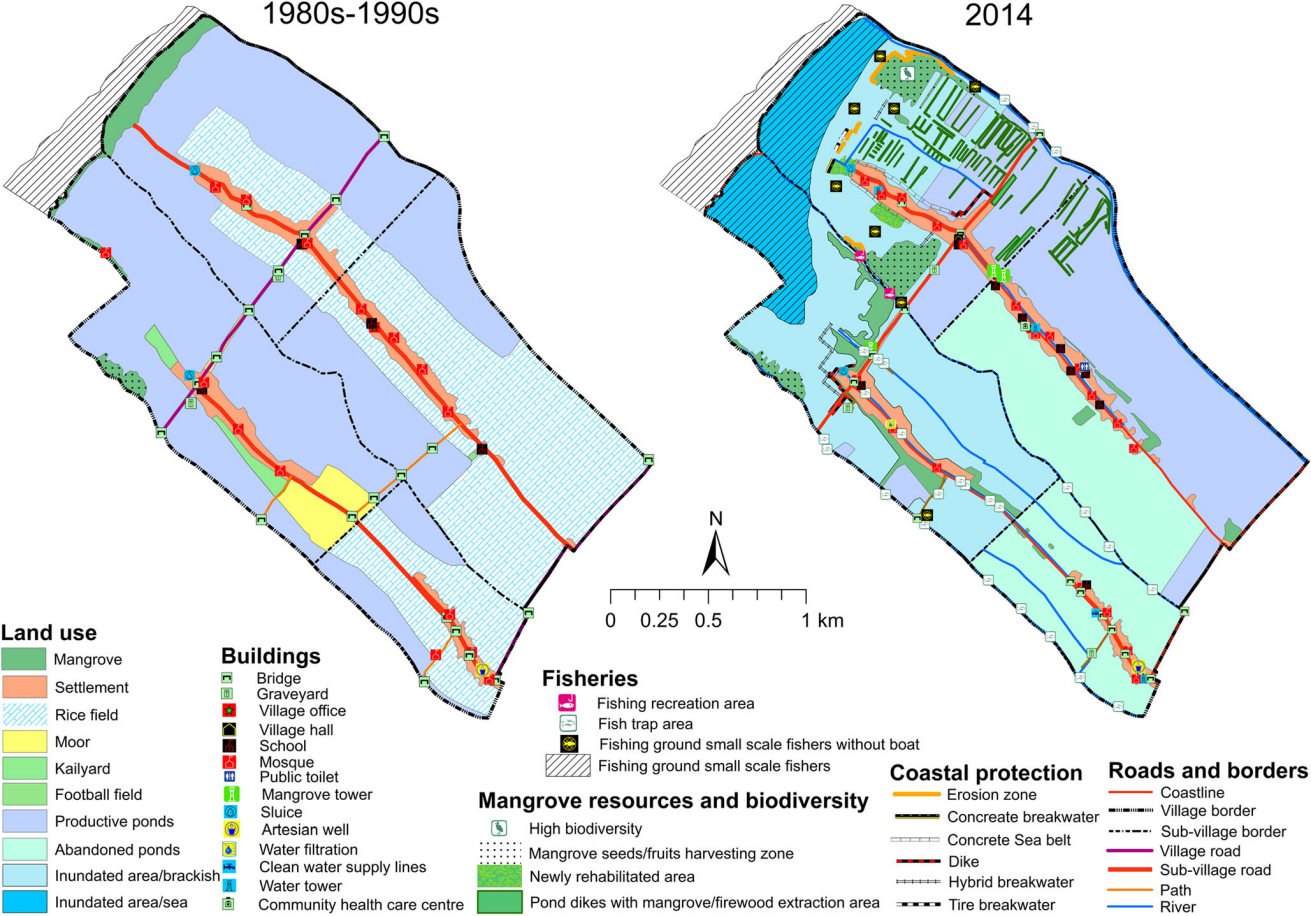
(left) Historical map (1980s to 1990s) and (right) present map (2014) of Timbulsloko

Similar to Bedono, Timbulsloko was also an agrarian village. The participants stated that their village was “*gemah ripah loh jinawi*”: peaceful, prosperous, and very fertile. In the 1980s and 1990s, the villagers were mostly rice and/or aquaculture farmers. Different from Bedono, some participants in Timbulsloko witnessed the presence of mangroves on the seashore in the 1980s. This testimony was connected to their childhood experience when they spent their time around the seaside (see Fig. 3, left). According to the participants, the mangroves slowly disappeared mainly due to increasing intensity of coastal erosion that started in the 1990s. In 2014, most of the rice fields, moors, and kailyards around the settlement had gone (see Fig. 3, right). Some of them were inundated, and the rest was converted into aquaculture. Likewise, more than half of the ponds were flooded and abandoned by the owners. Nevertheless, most of the ponds located in the North Eastern part of the village (Karanggeneng and Wonorejo Sub-Villages) were less impacted and still productive.

Similar to Bedono, various national and regional mangrove rehabilitation programs have been implemented in this village since 1999 by different actors, including Demak Environmental Office, Demak Agricultural Office, Demak Marine, and Fisheries Office, NGO, Educational institutions, and private sectors. NGO involvement only started in 2013 through a project (“Mangrove Capital”) led by Wetlands International. In 2014, OISCA also began to set up mangrove planting projects in this village. Aside from the conservation objective, rehabilitation also aimed to protect the village from erosion, flooding, and inundation. Other coastal protection measures were also mentioned during the mapping process, including construction of tire, concrete, and hybrid-breakwater and sea-belt as shown in Fig. 3 (right).

### Changes of locally important mangrove ES

**Fig. 4:**
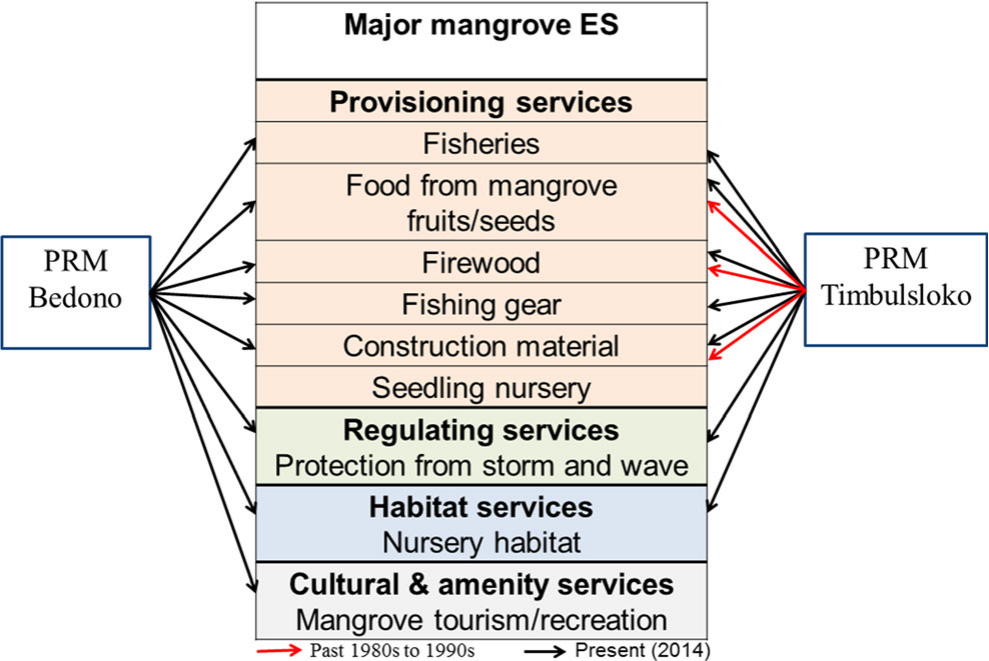
Major ES identified by PRM participants

The rehabilitation executed in the two villages has been able to restore the lost or degrading mangrove ecosystem services, i.e., provisioning, regulating, habitat, and cultural and amenity services. Our finding shows increasing communities’ dependency on these services through the increasing number of services used or perceived as important by the participants (Fig. 4).

#### Provisioning services

During the mapping process, the participants in both villages recalled three primary mangrove ecosystem services utilized by communities, especially before the 1980s. These services include food, firewood, and construction material. Before the mangroves disappeared, the dominant species recognized by the participants in both villages was *Avicennia marina* or locally called brayo. Local communities used to harvest brayo for food, either as complementary or substitute of staple food. Another type of utilization mentioned was firewood extraction. In the past, firewood was the most important source of energy beside kerosene. Therefore, extracting firewood from mangroves reduced expenditures needed for energy used in cooking activities. Additionally, the participants also identified mangrove utilization for pond construction notably to regulate the water in the canal around the ponds. This information showed a relatively low communities’ dependence on mangrove ES. Therefore mangrove conversion to aquaculture ponds for higher economic benefits was more favorable for most of the villagers rather than conservation to sustain mangrove ES. This attitude, according to the participants, was caused by a lack of knowledge on the benefits provided by these ecosystems which only became apparent after the mangroves were lost.

The return of mangroves in the two villages changed the communities’ attitude toward these ecosystems. This is shown among others by the increasing variety of resource utilization mentioned by participants. They can now harvest *Avicennia marina*’s seed again, mainly for cultural and seasonal complementary foods. The participants in both villages also mentioned the use of mangroves for firewood. In Bedono, the firewood was extracted from the mangroves on the seaside, whereas in Timbulsloko, this resource was mainly extracted from the mangroves around the ponds which are only accessible for the pond owners. Likewise, construction material, particularly mentioned in Timbulsloko, was only extracted from privately owned pond areas.

In the past, mud-crab had a very low economic value. Therefore, the communities only caught this species for household consumption. Since the late 1990s, mud-crabs became a valuable export commodity and the price in the local market has increased substantially, up until recently. The participants recognized that the mud-crab abundance increased after the rehabilitation. This phenomenon has raised the number of fishers specialized in catching this species. Many of these fishers used mud-crabs as their primary source of income. According to the participants, the mud-crabs fishing activities were conducted using different tools (i.e., crab finders, crab traps, crab lines, crab nets) that can be associated with the fishing ground. The crab finder is a long iron rod with a hooked tip that is mainly used to catch the crabs burrowing underneath *Rhizophora*’s roots. Activities using this gear are usually conducted within the mangrove areas. The crab traps are commonly placed in the creek around the mangrove areas. One fisher often used dozens of mud-crab traps when fishing. Lastly, crab lines and crab nets are used to catch the crabs in different locations around the mangrove areas.

Two other types of resource utilization that was not historically used are the extraction of fishing gear and seedling nursery. As mentioned in “Spatial and narrative construction of the villages’ previous and present condition,” most of the villagers in Bedono and Timbulsloko were previously farmers whose livelihood depended on natural assets (i.e., rice fields, moors, kailyard, and ponds). The recent occupational transition to fishers in the two villages was mainly caused by the continuous erosion and inundation that damaged and even destroyed these natural assets. Consequently, rising number of fishers increased mangrove utilization for fishing gear such as fishing rode or stakes for fish traps. Furthermore, the harvesting of mangrove seedlings was mentioned as an additional source of livelihood that emerged along with the expanding mangrove areas and rehabilitation projects implemented by different institutions within and outside the district. This small-scale business is mainly organized by community associations, and based on pre-ordering. The primary species used for this business is *Rhizophora sp* harvested from mangrove areas on the seaside.

#### Regulating services

Mangrove regulating services, particularly coastal protection from storms and damaging waves, was strongly addressed when constructing the present map. All participants in Bedono and Timbulsloko had experienced the impact of various coastal hazards before and after the presence of mangroves in their villages. The participants confirmed the decreasing impact of wave and storm damage since the mangroves grew around their houses. This experience increased their knowledge on the importance of mangroves to protect their villages. They are currently worried about the constant erosion that threatens the sustainability of the rehabilitated mangrove ecosystems. Therefore, they marked the mangrove areas that are prone to erosion as guidance for further management discussion.

#### Habitat services and biodiversity hotspots

While constructing the map of present conditions in both villages, the participants recognized the correlation between some commercially important fish species, i.e., mud-crabs (*Scylla sp.*), white shrimp (*Penaeus merguensis*), and the return of mangroves in their villages. They stated that the larger the mangrove area, the higher the abundance of these species. This statement shows an increasing understanding of mangrove importance to fisheries based on their experience or observation. Additionally, to improve fisheries, the participants also recognized that the increasing size of the rehabilitated areas also increased the number of bird species in their villages. Therefore during the mapping process, they also marked the areas with high bird diversity as biodiversity hotspots.

#### Cultural and amenity services

The participants mentioned two mangrove recreation sites in Tambaksari sub-village and Rejosari sub-village, Bedono. The recreation site in Tambaksari is more popular than the one in Rejosari due to the presence of a sacred tomb. Most of the visitors visited this area for spiritual pilgrimage. However, many visitors also visit the areas to enjoy the beautiful mangrove scenery.

### Enhancement of local mangrove management

When we started the mapping exercise, the only spatial information we found in Bedono was a hand-drawn map created in 1997 while in Timbulslokoa scaled map of village development scenarios, created by academics from a local university, existed but was kept in the house of the village headman. Spatial information on land use, resources, and redeveloped mangrove areas, thus did not exist in both villages. By participating in the mapping activities, villagers gained valuable new insights on the village’s potential, for example, fishery and tourism, and identify potential areas for rehabilitation of mangrove areas prone to erosion (pers. Comm. Nurrochman, Sifatul khoiriyah, Suratno, Nadhiri). Several villagers stated that the spatial data provided valuable information to understand the landscape changes, enhance current mangrove management programs and develop villager’s potential to formulate mitigation and adaptation plans to deal with the environmental degradation that threatens these villages.

Among the most important phenomena observed during the mapping process was the increasing confidence of the participants in communicating their ideas, opinions, and management planning to the government and NGO representatives. They confidently express their knowledge of the features shown on the map and asked for the support from the government and NGO to provide them with necessary assistance to improve mangrove and village development. Such a bottom-up communication process had never happened before and the government and NGO representatives responded positively by setting up follow-up discussions and stimulated the participants to use the maps as one of their communication tools.

## Discussion

Participatory mapping is increasingly applied in ES assessment (Brown and Fagerholm 2015; Paudyal et al. 2015; Ramirez-Gomez et al. 2015). Using participatory resource mapping (PRM), we were able to identify the major ESs provided by the rehabilitated mangrove ecosystems in the study area based on local perception. This participatory approach also enabled us to reconstruct spatial information on the village history back in the 1980s and 1990s to understand the changes in the landscape and mangrove ES using collective memories. However, there are many limitations and uncertainties involved in this approach which are discussed in this section.

### Suitability of PRM to identify ES

Brown and Fagerholm (2015) point out that participatory mapping is suitable to identify provisioning and cultural benefits that are grounded in personal experience. Studies using this method show that participants are eager to identify provisioning and cultural services, but are quite challenged to identify regulating and habitat services (Brown and Fagerholm 2015). Interestingly, in our study, we observed the opposite phenomenon. The participants were able to readily identify mangrove regulating services, particularly coastal protection, when constructing the present map, but were reluctant to identify some provisioning services. This opposite finding was probably due to participants’ experience related to coastal hazards and the presence of village laws that regulate mangrove utilization and protection.

#### Influence of participants personal experience

All participants had experienced the decreasing impact of storms and waves since the mangroves returned to their villages. This collective experience influenced their perception on the importance of mangroves to protect their village from coastal disasters. However, they realized that the mangroves are also threatened by continuous coastal erosion, which raised their concern to protect these rehabilitated ecosystems. This probably influenced their attitude when discussing the main benefits derived from the presence of mangroves in their villages. During the mapping process, the participants had difficulty in identifying other types of regulating services such as nutrient recycling or carbon sequestration. However, they understood the positive correlation between larger mangrove areas and the increasing abundance of crustacean species, particularly *Scylla sp* and *Penaeus merguensis* based on their experience*.* This reflects their understanding of the nursery service provided by mangrove ecosystems.

#### Influence of existing regulations and norms

All the rehabilitated mangroves are protected under local regulation. The participants recognized the village law that forbids mangrove cutting or logging and is aware of the sanctions. We realized that disclosing such information can potentially exacerbate tension among the participants. Therefore, when facilitating the discussion, we refer directly to the types of goods that can be extracted from the mangroves without causing any destruction. However, it is important to notice that there may be other types of utilization that were not mentioned during the mapping process due to the sensitivity of the matter. In this case, more personal approach such as open-ended, semi-structured, or questionnaire-based interview is needed to complete the information. These findings show that the completeness of ES data collected through workshop-based PRM is influenced by existing rules or norms, participants’ experience, and by conflicting interests.

### Data quality, accuracy, and precision

According to Brown and Kyttä (2014), the quality of information generated through participatory mapping can be evaluated using two indicators, sufficiency of spatial data and the inclusion of stakeholders who have influence on and/or are affected by management decisions. Sufficient spatial data can be obtained through the inclusion of the most appropriate stakeholders (Brown and Fagerholm 2015; Opdam 2013). Likewise, the participants involved in the participatory mapping process must be accompanied by sufficient spatial data (Brown and Kyttä 2014).

#### Selection of stakeholders

In our approach, we used purposive sampling to ensure that all relevant stakeholders who influence or affected by mangrove rehabilitation and management are included. This sampling design has been widely used to conduct mapping activities through workshops or focus groups within the rural setting of developing countries (Brown et al. 2014) like Indonesia. In our study, we selected the participants based on consultation with the village authority and own observations after informal communication with villagers. The consultation was necessary to follow local procedures and to build cooperation with village governments (Ramirez-Gomez et al. 2013). However, independent observation of the local political situation and potentially important participants are required to ensure stakeholder representativeness. This is also important to avoid that the ruling authority selects participants based on their political interests (Berbés-Blázquez 2012; Meilasari-Sugiana 2012).

#### Availability of spatial data

Regarding the spatial data, we used non-digital base maps to accompany the participants in the field. The non-digital map is culturally more suitable and practical for mapping in rural areas compared to, for example, digital maps (Brown and Fagerholm 2015). As stated by Brown and Kyttä (2014) problems related to data sufficiency commonly occurs when dealing with larger study areas. Our study covers only a small area, therefore, village satellite imagery retrieved from Google Earth in 2014 was sufficiently suitable since it provides detailed spatial information at the village level. The participants also stated that they could understand the base map used in the mapping process.

#### Accuracy and precision

Participatory mapping applied in rural areas of developing countries often aims for social learning, conflict resolution, or building social capital by engaging non-experts of a society. Therefore, the participation component is often perceived as more important than precision and accuracy.This raised questions about the reliability of the data. Precision refers to the exactness in placing the markers on the base map, whereas accuracy reflects the closeness of the markers to the spatial dimensions of the attribute being mapped (Brown and Kyttä 2014; Vajjhala 2005). In our study, we asked the participants to mark the attributes on the base map in the form of colored areas with additional descriptions written on sticky notes. The base map was then used as guidance during the ground truthing with participants where they marked the attributes using GPS. This process aimed at collecting accurate and precise geo-referenced information of the attributes being mapped.

Regarding the output, we found many similarities between the landscape changes perceived by participants and the remote sensing images of the two villages collected by Wetlands International (WI), an International NGO currently works in the studied area. The remote sensing images show mangrove conversion to aquacultures during the period 1988–1989. The mangroves in the two villages continuously decreased and most of the agricultural land had been converted into ponds by the end of the 1990s. The earliest erosion was identified in Bedono village in 1996, and the size of mangrove areas in the two villages continuously increased since 2005. The comparison suggests that the remote sensing images provide an accurate picture of the historic land cover change (Ariti et al. 2015), whereas the PRM provided detailed narratives on the causes of the changes which are essential for management. The narratives of the past situation were based on the consensual agreement between participants involved in the mapping activities. We therefore assumed that uncertainty might exist, especially related to the accuracy of the mapped features and their narratives, i.e., the exact date, the exact location, etc. Furthermore, we also realized that our findings are insufficient for regional generalization (Brown and Kyttä 2014; Ramirez-Gomez et al. 2015) due to limited spatial information generated from the mapping activities. Our study should therefore be considered as the first step of a more detailed ecosystem services assessment that needs further work to obtain sufficient information for decision support at the regional level.

### Facilitation and ethical issues

Many studies on participatory research showed that the manner in which the participatory process is conducted has more influence on the outcome than the tools that are used (Chess and Purcell 1999; Reed 2008; Richards et al. 2004). Different facilitators with different levels of facilitation skills could generate different outcomes even if they applied similar tools (Reed 2008). In our study, we applied several techniques to aid the facilitation process as suggested by some studies. These techniques include (1) observation of the local social and political situation through personal and informal contact prior and in between the mapping process; (2) the use of local language to avoid miss-understanding; (3) development of ground rules agreed by all participants; (4) meticulous planning and; (5) encourage participants to question and state their opinion (Berbés-Blázquez 2012; Chambers 2006; Chess and Purcell 1999; Rambaldi et al. 2006a; Reed 2008; Richards et al. 2004).

Another aspect to be aware of is the fact that the concept of ES is rooted in a particular tradition of western science (Berbés-Blázquez 2012) which was not known by the participants. Therefore, this concept was adapted to the local context by using other terms such as resources, benefits, functions, and sometimes directly referring to the types of goods. We realized that high facilitation skills (e.g., handling conflict, dealing with dominating and offensive individuals, maintain positive group dynamics) are challenging. These skills tend to develop through years of practices and experience (Reed 2008; Richards et al. 2004). However, meticulous preparation and observation of local culture and politics, grounded communication strategy and facilitation technique using local language enabled us to collect sufficient spatial information required for this study.

The application of participatory mapping often raises ethical issues such as taking people’s time, raising expectations, and knowledge extraction for the benefit of the outsiders (Chambers 2006; Rambaldi et al. 2006a). Therefore, clear communication of the expected outcomes is necessary (Brown and Fagerholm 2015). During the introduction meeting, we explained, in the local language, our background, objective, and output of the mapping activities. We provided information on various participatory mapping methods and steps, and examples of the output. We emphasized voluntary participation and consensus among participants. The participants were also given the freedom to select the methods and steps suitable for them and arrange the schedule for the mapping activities. Through this process, they were fully aware of their role, the expected output and outcomes and time that will be asked from them. However, as stated by Chambers (2006) and Rambaldi et al. (2006a), time for rural people, including in the two villages, is very precious, yet they are polite, hospitable, and differential to researchers or strangers who are often unaware of their sacrifice. Most of the participants, particularly farmers, fishers, or laborers, often get their income on a daily basis. A day off also means losing a substantial amount of money that support their household. Therefore, most of the activities were held during the weekend. Additionally, we also provided sufficient financial compensation for the activities conducted during the participants’ working hours.

A common problem in participatory research is the extraction of various forms of local knowledge for the benefit of the outsiders or researchers without being clear to those who provide the information. Chambers (2006) and Rambaldi et al. (2006a) provide examples of villagers in Malawi who were asked to map their village by outsiders repetitively, and the results were always taken away by the outsiders. Such situations not only exploit local communities, but also leaves the communities in a powerless situation. Therefore, in this study, we realized the ethical necessity to ensure that the output resulted from the mapping process can be understood by all stakeholders and that the result is given back to the participants. Therefore we involved the participants and other related stakeholders to evaluate the resulting maps. This step is vital to ensure that all attributes were included in the maps and that all stakeholders understand the output. Furthermore, we also disseminated the physical map to the participants and the digital version to other stakeholders involved in mangrove management in the final stage of the mapping process.

### Data usability and impact to mangrove management

Production of spatial information that influences land-use decisions and ecosystem management are important objectives of participatory processes. However, as highlighted by Brown and Fagerholm (2015), social learning and creation of social capital are also equally important objectives to achieve sustainable land use. In this study, we could verify the achievement of these objectives by observing the group dynamic and communication between different stakeholders during the mapping process, and discussion and interview with participants and other relevant stakeholders on the benefits derived from the mapping process and output.

Based on our observation, the involvement of different representatives of the communities in the PRM process enable the exchange of knowledge between participants and thus facilitate social learning among them. Likewise, the result socialization and dissemination workshop, facilitate the dialog between different stakeholders, i.e., communities, government, NGOs, and academics, to better manage the rehabilitated ecosystems using the resulted map as their baseline information. The learning process during the mapping exercise and the resulted map equipped the participants with sufficient knowledge and tool that boosted their confidence when communicating their opinion and management plan to other stakeholders. This phenomenon showed that the mapping process provided the foundation for the creation of social capital. Although, it is still too early to tell about the influence of our study to the policy related to mangrove management. The whole mapping process aspires to be the initial step in influencing and enhancing local mangrove management to achieve a sustainable result.

## Conclusion

Our study shows that workshop-based PRM can elicit consensual information on mangrove ES, their changes over time and space, and the factors influencing the ES changes. The results showed the difference in actual use and perception of villagers of mangrove services in the past (before they were all destroyed or severely degraded) and after rehabilitation. By applying PRM combined with GIS, we were able to collect spatial information on different mangrove resources and ecosystem services utilized by communities and identify key areas including harvesting zones, biodiversity hotspots, erosion zones, fishing grounds for the different type of fishers, and newly rehabilitated areas. The results also show that the return of mangroves has changed communities’ attitude toward these ecosystems. The types of mangrove resources utilized by the communities are currently more diverse compared to pre-rehabilitation times. Due to their participation in the mapping exercise, the communities are more aware of the importance of the rehabilitated ecosystems to protect their villages from coastal hazards. This information can be used as valuable baseline information for management and (economic) valuation. However, application of workshop-based PRM can also lead to conflicts among participants, especially when it reveals sensitive information related to activities that potentially violate local rules or norms. Furthermore, PRM can potentially disempower communities when the potential ethical implications of this method are neglected. Meticulous planning, good facilitation, and ownership sharing of the results are therefore essential when conducting PRM. Despite these constraints, our results show that by involving local communities from the beginning, participatory ES mapping can facilitate social learning, provide the foundation for the creation of social capital and equip the community with sufficient spatial information to improve local mangrove management. We believe that our “inclusive participatory ES mapping approach” can be used as a model to support local and regional decision-making processes and to enhance community-based mangrove management in other coastal regions in Indonesia and beyond.
